# Cross-Cultural Adaptation and Validation of the Dutch Version of the Hip and Groin Outcome Score (HAGOS-NL)

**DOI:** 10.1371/journal.pone.0148119

**Published:** 2016-01-28

**Authors:** Erwin Brans, Joost S. de Graaf, Arvid V. E. Munzebrock, Bram Bessem, Inge H. F. Reininga

**Affiliations:** 1 Department of Surgery, Medical Center Leeuwarden, Leeuwarden, The Netherlands; 2 Department of Trauma Surgery, University Medical Center Groningen, Groningen, The Netherlands; 3 Center for Sports Medicine, University Medical Center Groningen, Groningen The Netherlands; University of Michigan, UNITED STATES

## Abstract

**Background:**

Valid and reliable questionnaires to assess hip and groin pain are lacking. The Hip and Groin Outcome Score (HAGOS) is a valid and reliable self-reported measure to assess symptoms, activity limitations, participation restrictions and quality of life of persons with hip and/or groin complaints. The purpose of this study was to translate and cross-culturally adapt the HAGOS into Dutch (HAGOS-NL), and to evaluate its internal consistency, validity and reliability.

**Methods:**

Translation and cross-cultural adaption of the Dutch version of the HAGOS (HAGOS-NL) was performed according to international guidelines. The study population consisted of 178 adult patients who had undergone groin hernia repair surgery in the previous year. All respondents filled in the HAGOS-NL, the SF-36, and the SMFA-NL for determining construct validity of the HAGOS-NL. To determine reliability, 81 respondents filled in the HAGOS-NL after a time interval of two weeks.

**Results:**

Factor analysis confirmed the original six-factor solution of the HAGOS. Internal consistency was good for all the subscales of the HAGOS-NL. High correlations were observed between the HAGOS-NL and the SF-36 and SMFA-NL, indicating good construct validity. The HAGOS-NL showed high reliability, except for the subscale *Participation in Physical Activities* which was moderate.

**Conclusions:**

The HAGOS was successfully translated and cross-culturally adapted from English into Dutch (HAGOS-NL). This study shows that the HAGOS-NL is a valid and reliable instrument for the assessment of functional status and health-related quality of life in patients with groin complaints.

## Introduction

With 30,000 people treated annually, inguinal hernia repair is the most common operation performed in general surgery in the Netherlands [1]. In the past, recurrence rate has been used as primary outcome measure following inguinal hernia repair. Due to improvement of surgical techniques, outcome assessment shifted from recurrence rate to adverse outcomes of hernia surgery [2]. Persistent groin pain following inguinal hernia repair is a highly underestimated complication. Percentages up to 63% have been reported at 1 year after surgery [3].

Groin pain affects both physical functioning and health-related quality of life [2, 4]. Because groin pain is difficult to assess objectively, the use of Patient-Reported Outcome Measures (PROMs) in the evaluation of groin hernia repair has been advocated by numerous authors [2, 5–8]. The routine use of PROMs before and after receiving groin hernia repair surgery was introduced into the National Health Service (NHS) England in 2009 [9]. Generic PROMs are used by the NHS because reliable disease-specific PROMs to measure physical functioning and health-related quality of life in patients with groin complaints are lacking [9].

To date, there are a few hernia-specific questionnaires, such as the Inguinal Pain Questionnaire (IPQ) [10], Ventral Hernia Pain Questionnaire (VHPQ) [11] and Core Outcome Measures Index for inguinal hernia patients (COMI-hernia) [6]. However both the IPQ and VHPQ address only groin pain, but they do not assess the effect of groin disability on physical functioning and health-related quality of life. Although the COMI-hernia questionnaire consists of several underlying constructs, a total score is calculated for the COMI-hernia. Hence, valuable information about these different constructs might be lost.

The Copenhagen Hip and Groin Outcome Score (HAGOS) was therefore developed by Thorborg et al. [12] to assess pain, physical functioning and quality of life in patients with hip and/or groin pain. A Danish, Swedish and an English version of the HAGOS is available [12, 13].

To our knowledge there is no validated Dutch PROM available to assess groin complaints. Aim of this study was to translate and cross-culturally adapt the HAGOS into Dutch, and to evaluate the clinimetric properties of the Dutch version of the HAGOS in terms of internal consistency, validity and reliability.

## Methods

### Translation procedure

The HAGOS was translated into Dutch according to guidelines proposed by Beaton et al. [14] using six steps: Step 1: Two bilingual translators with Dutch as their mother tongue independently translated the English version of the HAGOS into Dutch (forward translation). Step 2: The translators and a recording observer synthesized the two forward translations in one forward translation. Differences were resolved by consensus. Step 3: Two translators whose native language was English and were fluent in Dutch translated the forward translation of the HAGOS back to English. Both were uninformed of the concepts explored, and were blinded to the original HAGOS. Step 4: An expert committee consisting of the forward and backward translators, a sports medicine physician and a clinical epidemiologist constructed the pre-final version of the Dutch HAGOS (HAGOS-NL). The synthesis process was carefully documented and differences were resolved by consensus. This resulted in the pre-final version of the HAGOS-NL. Step 5: The pre-final version of the HAGOS-NL was tested in a pilot, consisting of 20 patients, who were suffering from groin complaints. The patients were interviewed about the comprehension of items and the chosen response. Step 6: The final version of the HAGOS-NL was realised and evaluation of clinimetric properties of the HAGOS-NL was carried out in terms of internal consistency, validity and reliability.

### Participants

A total of 373 groin hernia repairs were performed on adult patients between October 2011 and September 2012 at the Medical Center Leeuwarden (MCL). Patients aged 70 years and older were excluded, since these patients may differ substantially from younger patients in terms of physical activity, functional limitations and co-morbidity. Patients who did not understand the Dutch language were also excluded, leaving 293 eligible patients.

**Fig 1:**
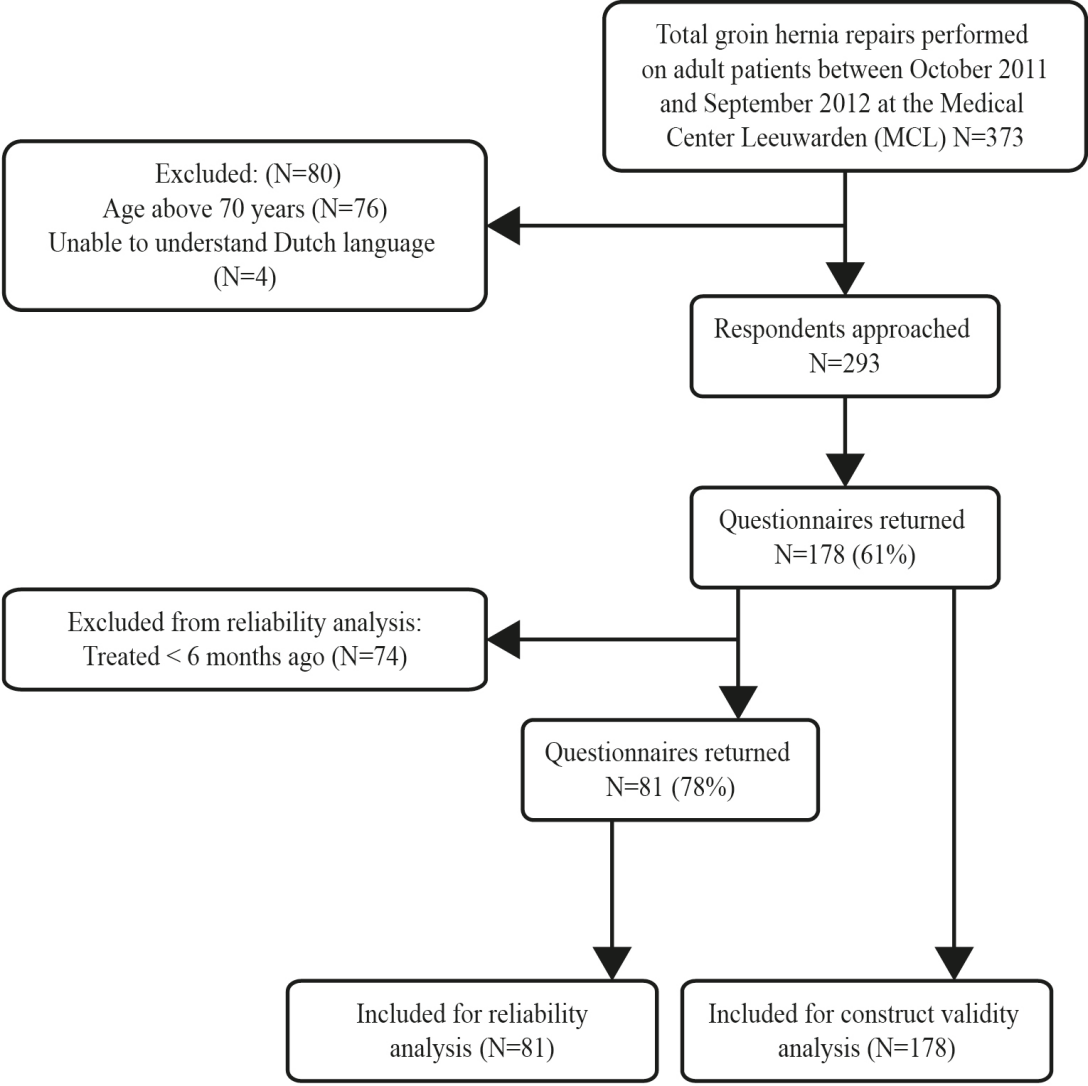
Flow chart for inclusion procedure of respondents.

The participants were contacted by mail. Participants were asked to fill in three questionnaires, namely the HAGOS-NL, the Short-Form 36 (SF-36) [15] and the Dutch Short Musculoskeletal Function Assessment (SFMA-NL) [16]. To evaluate reliability of the HAGOS-NL a test-retest procedure was used. Patients who were treated at least 6 months before were assumed to be in a stable physical state and were asked to fill in de HAGOS-NL again after two weeks. The flow chart of the inclusion procedure of respondents is presented in Fig 1. The study was approved by the medical ethics committee of the University Medical Center Groningen. No specific consent form was required as the ethics approval stated that completion of the quesionnaires could be taken as implied consent: this was explained to participants in the participant information sheet.

### Measures

The Hip and Groin Outcome Score (HAGOS) [12] is a disease-specific health questionnaire for people who are suffering from hip and/or groin complaints. The HAGOS was constructed and validated according to the Consensus-Based Standards for the Selection of Health Status Measurement Instruments (COSMIN) checklist [17]. The HAGOS consists of 36 items divided into six subscales, assessing: *Symptoms*, *Pain*, *Physical function in daily living*, *Physical function in Sport and Recreation*, *Participation in Physical Activities* and *Hip and/or groin-related Quality of life*. All items are scored on a 5-item Likert scale, ranging from 0 (no symptoms) to 4 (extreme symptoms). Scores for the individual subscales are calculated by summing the scores of individual items and transforming them to a scale from 0 to 100, with a higher score indicating a better function.

According to the validation of the original HAGOS, the HAGOS-NL was compared with the Short-Form 36 (SF-36) [15]. The SF-36 is a widely used generic health questionnaire to measure a person’s health status. The HAGOS-NL was also compared with the SMFA-NL [16], a questionnaire developed for use in the management of patients with a broad range of musculoskeletal injuries or disorders.

### Statistical analysis

The sample size used followed the recommendations of the COSMIN checklist [17], which states that the sample size for internal consistency should be ≥100 respondents and 5–7 times the number of items. The HAGOS consists of 37 items accross six subscales, based on the COSMIN recommendations, a sample size should contain least 185 respondents. All returned HAGOS-NL questionnaires were used for internal consistency analysis (n = 259), i.e. those used for assessing construct validity (n = 178) and reliability (n = 81). A good sample size for reliability (including test-retest reliability) is considered 50–99 patients [17]. To verify the reliability of the HAGOS-NL the current study had 81 respondents included in the reliability analysis.

All statistical analyses were performed using SPSS 20.0 for Windows (SPSS Inc, Chicago, USA). A p-value <0.05 was considered to indicate statistical significance. Missing data were treated according to the guidelines proposed bij Thorborg et al. [12] up to two missing values are substituted with the average value for the scale. If more than 2 items were missing, the response was considered invalid and no total score could be calculated for the subscale. If more than 1 item was missing for the *Participation in Physical Activities* scale the score was also considered invalid. Missing items of the SF-36 and SMFA-NL were treated according to the guidelines proposed by the developers of the used questionnaires [18, 19].

### Internal consistency

Internal consistency is a measure of homogeneity between a set of items [20]. Homogeneity refers to the extent to which items measure the same construct, and thus form a subscale. Exploratory factor analysis can be performed to uncover the underlying structure of the individual items. In the case of a predefined factor structure, confirmatory factor analysis is recommended [21].

Confirmatory factor analysis was conducted on all the individual subscales of the HAGOS-NL using Principal Component Analysis with Varimax rotation. Cronbach’s Alpha was calculated for each subscale. A Cronbach’s Alpha value between 0.70 and 0.95 is generally considered to indicate good internal consistency [20].

### Floor and ceiling effects

The presence of floor and ceiling effects may influence the validity and reliability of an instrument. Floor and ceiling effects are defined as 15% of the participants achieving the minimum or maximum score, respectively [22].

### Validity

Because of the absence of a gold standard, validity of the HAGOS-NL was expressed in terms of construct validity, which refers to the extent to which scores on a particular measure relate to other measures, consistent with theoretically derived hypotheses concerning the constructs that are being measured [20]. Construct validity was determined by comparing the subscales of the HAGOS-NL with the subscales of the SF-36 and SMFA-NL. Spearman’s Rho correlation coefficients were calculated between the subscales of the HAGOS-NL and subscales of the SF-36 and SMFA-NL. Spearman correlation coefficients were interpreted by Cohen: <0.30 = small; 0.30–0.50 = moderate; >0.50 = large [23].

Because the HAGOS was designed to measure physical function rather than mental and/or social function, the highest correlations were expected between the subscales of the HAGOS and the subscales of the SF-36 and SMFA-NL that are supposed to measure physical functioning (convergent validity) [24]. Smaller correlations were expected between the subscales of the HAGOS and the subscales of the SF-36 and SMFA-NL that are supposed to measure mental and/or social functioning (divergent validity) [24]. The highest correlations were expected between the subscales of the HAGOS-NL and the following subscales of the SF-36: *Physical function*, *Role limitations due to physical health problems* and *Bodily pain* (convergent validity). Smaller correlations were expected between the subscales of the HAGOS-NL and the following subscales of the SF-36: *General health perceptions*, *Vitality*, *Social functioning*, *Role limitations due to emotional problems* and *General mental health* (divergent validity). In general higher correlations were expected between the subscales of the HAGOS-NL and the following subscales of the SMFA-NL: *Lower-extremity dysfunction* and *Problems with daily activities* (convergent validity). Smaller correlations were expected between the subscales of the HAGOS-NL and the SMFA-NL subscale *Upper-extremity dysfunction*, because the HAGOS-NL only contains items concerning the lower extremities (divergent validity). Small correlations were also expected between the subscales of the HAGOS-NL and the SMFA-NL subscale *Mental and emotional problems* (divergent validity). A priori hypotheses were formulated (Table 1). If 75% or more of the arbitrarily set number of hypotheses were confirmed, the construct validity of the HAGOS-NL was considered good [20].

**Table 1:** A priori hypotheses concerning the correlations between the subscales of the HAGOS-NL and the subscales of the SF-36 and SMFA-NL ^a^.

	Confirmed yes/no
A correlation of at least 0.50 between the HAGOS-NL subscales *ADL* and *Sport/Recreation* and the SF-36 subscale *Physical functioning*.	yes
A correlation of at least 0.50 between the HAGOS-NL subscale *Pain* and the SF-36 subscale *Bodily pain*.	yes
A correlation of at least 0.40 between the HAGOS-NL subscale *Symptoms* and the SF-36 subscale *Bodily pain*.	yes
A correlation of at least 0.40 between the HAGOS-NL subscale *QOL* and the SF-36 subscale *Mental health*.	no
A correlation of at least 0.50 between the HAGOS-NL subscales *ADL* and *Sport/Recreation* and the SMFA-NL subscale *Lower-extremity dysfunction*.	yes
A correlation of at least 0.50 between the HAGOS-NL subscale *ADL* and the SMFA-NL subscale *Problems with daily activities*.	yes
A correlation of at least 0.40 between the HAGOS-NL subscale *QOL* and the SMFA-NL subscale *Mental and emotional problems*.	yes

^a^ Values are given as Spearman’s correlation coefficient.

ADL, Physical function in daily living; Sport/Recreation, Physical function in Sport and Recreation; QOL, Hip and or/groin-related Quality of Life.

### Reliability

Reliability concerns the degree in which an instrument can repeat identical measurements in stable patients. Intraclass Correlation Coefficients (ICCs) with the corresponding 95% confidence intervals (CI) were calculated for each subscale of the HAGOS-NL. The ICC two-way random effects model, type agreement, was used [25]. ICC values above 0.70 are considered an indication of high reliability [20].

With the Bland-Altman method [26] the mean difference between the first and second measurement is calculated, with its corresponding 95% CI. Zero lying in within the 95% CI is considered a criterion for absolute agreement. Hence, zero lying outside the 95% CI indicates a systematic bias between the first and second administration of the HAGOS-NL.

Additionally, the Standard Error of Measurement (SEM), an estimate of the size of absolute measurement error, was calculated. The value of SEM was calculated as σ√(1-ICC), in which σ represents the pooled SD of the first and second measurement [27]. The SEM was used to obtain the Smallest Detectable Change (SDC). The SDC at the individual level was calculated by 1.96 x SEM x √2, and at the group level by 1.96 x SEM x √2/√n [28].

## Results

### Descriptive statistics

The demographic characteristics of the respondents of de HAGOS-NL are shown in Table 2.

**Table 2:** Baseline characteristics of the patients who responded to the HAGOS-NL questionnaire.

Characteristics	HAGOS-NL^a^ (N = 178)
Age (years)	52 (14)
Gender	
Man	169 (95%)
Woman	9 (5%)
Marital status (N = 175)	
Single	33 (19%)
With partner	77 (44%)
With partner and children	61 (35%)
With children	4 (2%)
Educational level (N = 174)	
Elementary school	7 (4%)
High school	74 (43%)
College	48 (28%)
Bachelor’s degree or higher	45 (26%)
Procedure type	
Open	111 (62%)
Open (recurrence)	9 (5%)
Laparoscopic	52 (29%)
Laparoscopic (recurrence)	6 (3%)

^a^ HAGOS, Hip and Groin Outcome Score.

Values, except for age, are given as the number of patients. Age is given as mean (SD).

### Translation and cross-cultural adaptation

None of the 20 patients reported any problems during the completion of the pre-final version of the HAGOS-NL.

### Feasibility

At the first measurement 81 items (1.3%) of the HAGOS-NL were missing. There were 77 missing items on the SF-36 (1.2%) and 127 missing items on the SMFA-NL (1.6%). At the second measurement, 28 items of the HAGOS-NL were missing (1.0%).

### Internal consistency

We are using a predefined factor structure obtained bij Thorborg and his collegues [12]. Small improvements in Crohnbach’s alpha could be made by deleting *Symptoms* subscale question 2 and *Pain* subscale question 2 (see S1 Table). Because of the use of a predefined factor structure no items were dropped because of the inability to compare the results of our study with versions in other languages which contain all items.

Factor analysis confirmed the six-factor structure of the original HAGOS (Table 3). Cronbach’s Alpha of the individual subscales of the HAGOS-NL ranged from 0.90 to 0.98.

**Table 3:** Internal consistency measures of the subscales of the HAGOS-NL.

Subscale (N = 246)	Number of items	Eigenvalue	Variance explained	Cronbach’s Alpha
Symptoms	7	4.4	62%	0.90
Pain	10	6.9	68%	0.94
ADL	5	4.0	79%	0.93
Sport/Recreation	8	6.8	86%	0.98
PA	2	1.9	93%	0.92
QOL	5	4.0	79%	0.93

Abbreviations: HAGOS, Hip and Groin Outcome Score; ADL, Physical function in daily living; Sport/Recreation, Physical function in Sport and Recreation; PA, Participation in Physical Activities; QOL, Hip and or/groin-related Quality of Life.

### Floor and ceiling effects

Overall, no floor effects were seen in any of the subscales of the HAGOS-NL. However medium to large ceiling effects were seen in all subscales of the HAGOS-NL: *Symptoms* (24.3%), *Pain* (44.3%), *Physical function in daily living* (48.6%), *Physical function in Sport and Recreation* (45.8%), *Participation in Physical Activities* (48%) and *Hip and/or groin-related Quality of life* (32.6%).

### Construct validity

The correlations between the subscales of the HAGOS-NL, SF-36 and SMFA-NL are presented in Table 4. Large correlations (0.55 to 0.78) were seen between the subscales of the HAGOS-NL and the subscales of the SF-36 and SMFA-NL which are related to physical function, indicating good convergent validity. Small to moderate correlations (0.09 to 0.49) were found between the subscales of the HAGOS-NL and the subscales of the SF-36 and SMFA-NL which are related to the mental and/or social function, indicating good divergent validity. Of the predefined hypotheses, 86% could be confirmed. However, the correlation of 0.16 between the HAGOS-NL subscale *Hip and/or groin-related Quality of life* and the SF-36 subscale *Mental health* was lower than hypothesised.

**Table 4:** Spearman’s correlation coefficients between each subscale of the HAGOS-NL and SF-36 and SMFA-NL.

	HAGOS-NL					
	Symptoms	Pain	ADL	Sport/Recreation	PA	QOL
SF-36						
Physical Function	**0.59**	**0.61**	**0.67**	**0.59**	**0.61**	**0.62**
Physical Role	**0.57**	**0.55**	**0.58**	**0.59**	**0.60**	**0.60**
Bodily Pain	**0.68**	**0.69**	**0.73**	**0.69**	**0.68**	**0.78**
General Health	0.39	0.35	0.39	0.36	0.38	0.32
Vitality	0.28	0.21	0.22	0.19	0.30	0.27
Social Functioning	0.42	0.38	0.46	0.38	0.45	0.42
Emotional Role	0.33	0.32	0.34	0.28	0.37	0.35
Mental Health	0.18	0.09	0.09	0.11	0.22	0.16
SMFA-NL						
Lower-extremity dysfunction	**0.68**	**0.69**	**0.78**	**0.69**	**0.70**	**0.65**
Upper-extremity dysfunction	0.18	0.23	0.26	0.26	0.17	0.16
Problems with daily activities	**0.68**	**0.66**	**0.73**	**0.69**	**0.70**	**0.72**
Mental and emotional problems	0.47	0.41	0.49	0.44	0.49	0.41

Abbreviations: HAGOS, Hip and Groin Outcome Score; ADL, Physical function in daily living; Sport/Recreation, Physical function in Sport and Recreation; PA, Participation in Physical Activities; QOL, Hip and or/groin-related Quality of Life. Large correlations (>.50) are shown in bold, small (< .30) and moderate (0.30–0.50) correlations in regular text.

### Reliability

Reliability measures of the HAGOS-NL are shown in Table 5. ICC values were high (>0.70) for all the subscales of the HAGOS-NL, except for the subscale *Participation in Physical Activities*. The 95% confidence interval contained zero for all subscales, indicating no systematic bias between the first and second measurement. SEM values for the different subscales ranged from 8.0 (*Pain*) to 15.9 (*Participation in Physical Functioning*). The SDC at individual level ranged from 22.2 to 44.2 points, and at group level between 2.5 and 4.9 points for the different subscales of HAGOS-NL.

**Table 5:** Reliability measures of the HAGOS-NL.

Reliability (n = 79) HAGOS-NL	First measurement mean (SD)	Second measurement mean (SD)	Mean difference (95% CI)	SEM	SDC_ind_	SDC_grp_	ICC (95% CI)
Symptoms	83.1 (18.7)	82.9 (19.2)	0.2 (-2.6, 3.0)	8.9	24.6	2.8	0.78 (0.68, 0.86)
Pain	88.0 (18.1)	87.4 (17.7)	0.6 (-2.0, 3.1)	8.0	22.2	2.5	0.80 (0.70, 0.87)
ADL	86.2 (20.2)	85.6 (20.3)	0.6 (-2.6, 3.7)	9.9	27.5	3.1	0.76 (0.65, 0.84)
Sport/Rec (n = 74)	83.9 (24.3)	83.8 (24.5)	0.1 (-3.5, 3.8)	11.2	30.1	3.6	0.79 (0.69, 0.87)
PA (n = 80)	79.8 (26.3)	80.5 (26.1)	-0.6 (-5.7, 4.4)	15.9	44.2	4.9	0.63 (0.47, 0.74)
QOL (n = 81)	80.1 (22.3)	79.8 (23.3)	0.3 (-2.4, 3.0)	8.5	23.6	2.6	0.86 (0.79, 0.91)

Abbreviations: HAGOS, Hip and Groin Outcome Score; SEM, Standard Error of Measurement; SDC_ind_, Smallest Detectable Change at individual level; SDC_grp_, Smallest Detectable Change at group level; ICC, Intraclass Correlation Coefficient. ADL, Physical function in daily living; Sport/Rec, Physical function in Sport and Recreation; PA, Participation in Physical Activities; QOL, Hip and or/groin-related Quality of Life.

## Discussion

In this study, the Hip and Groin Outcome Score (HAGOS) was successfully translated and cross-culturally adapted into Dutch. The translated version of the HAGOS (HAGOS-NL) showed to be a valid and reliable questionnaire to assess functional status and health-related quality of life of patients after groin hernia repair.

Factor analysis was performed on all the individual subscales of the HAGOS-NL and confirmed the original six-factor structure. Internal consistency coefficients were high, ranging from 0.90 to 0.98, indicating good internal consistency of the subscales. These results are in line with those reported by Thorborg et al. [12] (Danish HAGOS) and Thomeé et al. [13] (Swedish HAGOS).

Overall, no floor effects were observed in any of the subscales of the HAGOS-NL. However, ceiling effects were present in all the HAGOS-NL subscales ranging from 24.3% to 48.0%. During the validation of the original HAGOS both ceiling effects up to 21.8% and floor effects up to 38.6% were reported at some point in time [12]. These differences in the presence of floor and ceiling effects may be due to different patient populations. During the validation of the original HAGOS, people were included during their time of treatment. In the current study, people were treated for their groin complaints in the previous year before participation in this study.

Construct validity of the HAGOS-NL was determined by comparing the subscales of the HAGOS-NL with the SF-36 and SMFA-NL. Six of the seven predefined hypotheses regarding the correlations between the HAGOS-NL and the SF-36 and SMFA-NL were confirmed, indication good construct validity [27]. One predefined hypothesis was not confirmed: the correlation between the HAGOS-NL *Hip and/or groin-related Quality of Life* subscale and the SF-36 *Mental health* subscale did not reach the expected 0.40 (0.17). This is in line with the validation study of the original HAGOS [12].

Reliability of the HAGOS-NL was good, except for the subscale *Participation in Physical Activities* which was moderate. The findings of the present study regarding reliability of the HAGOS-NL are in line with the reliability of the original HAGOS [12] and the Swedish HAGOS [13], except for the subscale *Participation in Physical Activities*. The moderate reliability of this subscale may be caused by the fact that this subscale contains only two items. Since the score on this subscale is converted to a 100-point scale, a small improvement/deterioration in groin complaints will cause a large difference in points at a 100-point scale. A subscale with less than three items is generally considered weak and unstable [29].

To date, there are a few hernia-specific questionnaires, such as the IPQ [10], VHPQ [11] and COMI-hernia [6]. However, these questionnaires are not frequently used as instruments to assess outcome following groin hernia. Moreover, the IPQ and VHPQ assess only pain, and not restrictions in physical functioning and health-related quality of life. The COMI-hernia questionnaire consist of 6 items regarding pain, function, symptom-specific well-being, general quality of life, and social and work disability [6]. Important information about these underlying constructs may be lost if only a total score can be calculated.

The HAGOS-NL could only partly be compared with these questionnaires in inguinal hernia patients [6, 10, 11]. The determination of reliability of the IPQ reported by Franneby et al. [10] could not be compared with the results of the HAGOS-NL, since for reliability of the IPQ, the kappa statistics, instead of ICCs were calculated. The HAGOS-NL showed similar reliability as the VHPQ [11] and COMI-hernia [6]. The validity of the IPQ, VHPQ and COMI-hernia was however not determined according to the Consensus-Based Standards for the Selection of Health Status Measurement Instruments (COSMIN) checklist [17], as no theoretically derived hypotheses concerning the constructs that are being measured were pre-defined.

Overall, the SEM and SDC values of the HAGOS-NL were comparable to the findings of Thorborg et al. [12] and Thomeé et al. [13]. The low SDC values at group level (between 2.5 and 4.9) strongly indicate that the HAGOS-NL is useful for group comparisons. However, SEM and SDC are distribution-based measures, with no information regarding what change is considered important by the patient. To determine if the SEM and SDC values are acceptable depends on what changes are minimally important on the HAGOS. SDC values should be smaller than the Minimal Important Change (MIC) to distinguish clinically important change from measurement error [20]. The MIC of the HAGOS has not yet been determined. Hence a future study is needed to evaluate responsiveness (sensitivity to change) and MIC of the HAGOS, which makes it a valid outcome measure after surgical hernia repair.

## Conclusions

The HAGOS was successfully translated and cross-culturally adapted into Dutch (HAGOS-NL). All subscales of the HAGOS-NL showed good internal consistency. Construct validity was good, since strong correlations were found between the HAGOS-NL and the SF-36 and SMFA-NL. The HAGOS-NL showed high reliability except for the subscale *Participation in Physical Activities*. This study showed that the HAGOS-NL is a valid and reliable instrument for the assessment of functional status and health-related quality of life of patients with groin complaints following inguinal hernia repair.

## Supporting Information

S1 Table Internal consistency measures of the subscales of the HAGOS-NL. (DOCX)
