# Supplementary material for: Cross-Cultural Adaptation and Validation of the Dutch Version of the Hip and Groin Outcome Score (HAGOS-NL)
Source: PLoS One. 2016 Jan 28;11(1):e0148119. doi: 10.1371/journal.pone.0148119 (PMC4731216; doi:10.1371/journal.pone.0148119)
Supplement: S1 Table — (DOCX) [file pone.0148119.s001.docx]

**S1 Table**

Internal consistency measures of the subscales of the HAGOS-NL.

| Subscale (N=246) | Number of items | Cronbach’s Alpha | Corrected item total correlation | Cronbach’s Alpha if item deleted |
| --- | --- | --- | --- | --- |
| Symptoms | 7 | 0.90 |  |  |
| S1 |  |  | 0.72 | 0.88 |
| S2 |  |  | 0.43 | **0.91** |
| S3 |  |  | 0.72 | 0.88 |
| S4 |  |  | 0.73 | 0.88 |
| S5 |  |  | 0.69 | 0.88 |
| S6 |  |  | 0.79 | 0.87 |
| S7 |  |  | 0.82 | 0.87 |
| Pain | 10 | 0.94 |  |  |
| P1 |  |  | 0.78 | 0.94 |
| P2 |  |  | 0.61 | **0.95** |
| P3 |  |  | 0.82 | 0.94 |
| P4 |  |  | 0.82 | 0.94 |
| P5 |  |  | 0.81 | 0.94 |
| P6 |  |  | 0.70 | 0.94 |
| P7 |  |  | 0.77 | 0.94 |
| P8 |  |  | 0.80 | 0.94 |
| P9 |  |  | 0.85 | 0.93 |
| P10 |  |  | 0.86 | 0.93 |
| ADL | 5 | 0.93 |  |  |
| A1 |  |  | 0.81 | 0.92 |
| A2 |  |  | 0.86 | 0.90 |
| A3 |  |  | 0.87 | 0.90 |
| A4 |  |  | 0.76 | 0.92 |
| A5 |  |  | 0.82 | 0.92 |
| Sport/Recreation | 8 | 0.98 |  |  |
| SP1 |  |  | 0.82 | 0.98 |
| SP2 |  |  | 0.92 | 0.97 |
| SP3 |  |  | 0.93 | 0.97 |
| SP4 |  |  | 0.88 | 0.97 |
| SP5 |  |  | 0.90 | 0.97 |
| SP6 |  |  | 0.92 | 0.97 |
| SP7 |  |  | 0.93 | 0.97 |
| SP8 |  |  | 0.91 | 0.97 |
| PA | 2 | 0.92 |  |  |
| PA1 |  |  | 0.85 | * |
| PA2 |  |  | 0.85 | * |
| QOL | 5 | 0.93 |  |  |
| Q1 |  |  | 0.83 | 0.92 |
| Q2 |  |  | 0.80 | 0.92 |
| Q3 |  |  | 0.85 | 0.91 |
| Q4 |  |  | 0.79 | 0.92 |
| Q5 |  |  | 0.87 | 0.90 |

Abbreviations: HAGOS, Hip and Groin Outcome Score; ADL, Physical function in daily living; Sport/Recreation, Physical function in Sport and Recreation; PA, Participation in Physical Activities; QOL, Hip and or/groin-related Quality of Life.

* Crohnbach’s alpha could not be computed.
